# Renal Biopsy: A much needed tool in patients with Systemic Lupus Erythematosis (SLE)

**DOI:** 10.12669/pjms.321.3386

**Published:** 2016

**Authors:** Sumbal Nasir Mahmood, Kunwer Naveed Mukhtar, Saima Deen, Faiza Nafees Khan

**Affiliations:** 1 Dr. Sumbal Nasir Mahmood, Associate Professor of Medicine, Diplomat American Board of Nephrology, Head, Department of Nephrology, Dr. Ziauddin University Hospital, Karachi, Pakistan; 2 Dr. Kunwer Naveed Mukhtar, Associate Professor of Medicine, Diplomat American Board of Nephrology, Head, Department of Nephrology, Liaquat National Hospital and Medical College, Karachi, Pakistan; 3 Dr. Saima Deen, MBBS, Resident Medical Officer, Department of Medicine, Dr. Ziauddin Medical University and Hospital Karachi, Pakistan; 4 Dr. Faiza Nafees Khan, MBBS, FCPS I, Fellow, Department of Nephrology, Dr. Ziauddin Medical University and Hospital Karachi, Pakistan

**Keywords:** Lupus Nephritis, Renal Biopsy, SLE, Proteinuria, Urinalysis

## Abstract

**Background and Objective:**

Systemic lupus erythematosis (SLE) is an inflammatory disorder associated with significant morbidity and mortality. Kidneys are frequently affected in SLE and various stages of lupus nephritis have been identified based on severity of the disease. Treatment varies with the staging and correct diagnosis is essential for timely intervention as it can have significant impact on morbidity and mortality. The objective of the study was to determine whether laboratory parameters of lupus nephritis (LN); including urinalysis, serum creatinine (S. Cr) and 24 hours urine protein can accurately predict histologic staging of the disease.

**Methods:**

This retrospective study was conducted in department of Nephrology, Liaquat National Hospital Karachi from January 2012 to December 2014. Fifty one patients of SLE who underwent renal biopsy were selected. Patients, urinalysis at the time of renal biopsy, serum creatinine and 24 hours urine collection for protein were noted. All patients renal biopsy was read by the same pathologist. Patients were clinically staged based on these parameters and their histologic staging based on biopsy findings were compared, to see their correlation. Data was analyzed using SPSS version 17. Chi-square test was used to analyze categorical data and p<0.05 was considered significant. Cohen’s kappa (κ) analysis was used to examine the agreement by comparing lupus nephritis staging done by laboratory and histological ground. P value <0.05 indicates that agreement was unlikely due to chance alone.

**Results:**

Among 51 patients analyzed, 37 patients were females (72.5%) and 14 patients were males (27.5%) with mean age of 32.51 + 16.91 years. In stage II, kappa (κ) of 0.304 represented fair strength of agreement and a p value of 0.012 (p<0.05)which was statistically significant. In stage III, kappa was 0.209 indicating none to slight agreement and a p value of 0.131 (p>0.05). In stage IV, kappa (κ) was 0.141 (slight agreement)and p value 0.301 (p>0.05) in stage V; kappa (κ) of 0.030 represented poor agreement and a p value of 0.828 (p>0.05).

**Conclusion:**

Staging of lupus nephritis done on basis of laboratory findings did not correlate well with underlying histological staging. Therefore, renal biopsy is an essential tool in approach to lupus nephritis in order to provide timely and appropriate treatment to patients.

## INTRODUCTION

Systemic lupus erythematosis (SLE) is an inflammatory disorder that affects virtually all organs of the body. It is associated with significant morbidity and mortality. Mainstay of treatment of SLE is based upon preventive measures as early detection can significantly change the outcome of the disease. Kidneys are frequently affected in SLE. The earliest manifestation of renal involvement being an abnormal urine sediment, with proteinuria being the most frequent abnormality.^1^ The renal function may be normal even with significant involvement histologically.^2^-^4^

The incidence of lupus nephritis is high in Asians ranging between 33%-55%.^5^,^6^ Most renal abnormalities emerge soon after diagnosis(usually within first 6-36 months).^1^,^5^,^7^ Renal involvement in SLE is diagnosed either with routine urinalysis, looking for protein and hematuria, an estimation of urine protein excretion and elevated serum creatinine. Confirmation is usually done by renal biopsy, as staging of the disease is important, since the treatment varies with the staging and clinical presentation may not accurately reflect the severity of histologic findings.^8^-^11^

We conducted this study to see if clinical renal parameters could predict the histologic staging and if renal biopsy could be avoided and treatment decision could be taken based on clinical grounds.

## METHODS

After informed consent and ethical committee approval of the hospital conducting the study, retrospective analysis of all patients with SLE that underwent renal biopsy between January 2012 and December 2014 was done. The study was conducted in department of Nephrology, Liaquat National Hospital Karachi. We studied 51 patients of lupus nephritis. SLE was diagnosed with a positive ANA and anti dsDNA. Patient’s demographic characteristics, urine analysis at the time of renal biopsy, serum creatinine and 24 hours urine collection for protein was noted. Patients clinical staging based on these parameters is shown in Table-I.

**Table-I:** Clinical Staging of Lupus Nephritis.

Stage	24 hoursProteinuria	Urine Analysis		Serum Creatinine

		Urine Protein	Urine Red Cells	
Stage I	<300mg	-	-	NL
Stage II	<1gm	+	-	NL
Stage III	>1gm	++	+	+/-
Stage IV	>1gm	++	++	+
Stage V	Nephrotic syndrome	+++	+/-	+
Stage VI	+	+/-	+/-	++

NL = Normal.

Urinalysis was done for the presence or absence of proteinuria, hematuria or both. Proteinuria was positive if urine dipstick was positive for protein, Hematuria was defined as RBC > 5 RBC/HPF. Serum creatinine of >1.2mg/dl was considered abnormal for females and creatinine of >l.4mg/dl was considered abnormal for males. 24 hours urine collection for protein was considered nonnephrotic if protein was 300- 2999 mg/24 hours in urine and > 3 gm /24 hrs protein was considered nephrotic. Staging based on Histology is shown in Table-II.

**Table-II:** World Health Organization (WHO) morphologic classification of lupus nephritis (modified in 1982).

*Class I: Normal glomeruli* a) Nil (by all techniques) b) Normal by light microscopy, but deposits by electron orimmunofluorescence microscopy
*Class II: Pure mesangial alterations (mesangiopathy)* a) Mesangial widening and/or mild hypercellularity (+) b) Moderate hypercellularity (++)
*Class III: Focal segmental glomerulonephritis (associated with mildor moderate mesangial alterations)* a) With “active” necrotizing lesions b) With “active” and sclerosing lesions c) With sclerosing lesions
*Class IV: Diffuse glomerulonephritis (severe mesangial, endocapillary, or mesangio-capillary proliferationand/or extensive subendothelial deposits)* a) Without segmental lesions b) With “active” necrotizing lesions c) With “active” and sclerosing lesions d) With sclerosing lesions
*Class V: Diffuse membranous glomerulonephritis* a) Pure membranous glomerulonephritis b) Associated with lesions of category II (a or b) c) Associated with lesions of category III (a-c) d) Associated with lesions of category IV (a-d)
*Class VI: Advanced sclerosing glomerulonephritis*

### Inclusion Criteria

All patients with SLE of both gender who underwent renal biopsy, and had serum creatinine, urinalysis and 24 hours urine protein measurements were included.

### Exclusion Criteria

Patients were excluded if SLE patients renal biopsy had inadequate specimen and diagnosis was inconclusive. In addition if any of the laboratory parameter was missing that patient was excluded from the study. Patients already on immunosuppressant or anti-proteinuric medication and patients who had other concomitant disease other than lupus nephritis on renal biopsy were also excluded.

### Statistical Method

Data were collected on pre designed Performa, and analyzed using SPSS version 17. Chi-square test was used to analyze categorical data and p<0.05 was considered significant. Cohen’s kappa (κ) analysis was used to examine the agreement between two diagnostic methods i.e. clinical parameter and histologic finding on renal biopsy with values ≤ 0 as indicating no agreement, 0.01–0.20 as none to slight, 0.21–0.40 as fair, 0.41– 0.60 as moderate, 0.61–0.80 as substantial, and 0.81–1.00 as almost perfect agreement. P value <0.05 indicates that agreement was unlikely due to chance alone.

## RESULTS

Total 51 patients were enrolled based on inclusion and exclusion criteria. Among 51 patients analyzed, 37 patients were females (72.5%) and 14 patients were males (27.5%) with mean age of 32.51± 16.91 years. Expected staging of lupus nephritis done on laboratory parameters showed 08 patients in stage II(15.6%), 14 patients in stage III (27.4%),20 patients in stage IV(39.2%), 9 patients in stage V(17.6%) and none of the patient was expected to have either stage I or VI on laboratory parameters. Histological classification of stages showed 3 patients of stage II(5.9%), 11patients of stage III(21.5%), 26 patient of stage IV (50.9%), 10 patient of stage V(19.6%) and 01 patient of stage VI(1.9%). No patient had stage 1 on renal biopsy. Twenty eight patients had renal insufficiency in our study (54.9%) while 23 patients (45.1%) had normal renal function. Minimum proteinuria was found to be 72mg/24hr and maximum proteinuria was 8000mg/24hr.

We applied kappa analysis to measure agreement by comparing lupus nephritis staging done by laboratory and histological ground. The level of agreement between diagnostic methods indicates level of accuracy of diagnosis. Results are shown in Table-III.

**Table-III:** Results comparing two diagnostic methods.

		Histological Staging						
	Stage		Pos	Neg	Total	Kappa	95% CI	P value
ClinicalStaging	II	Pos	2	6	8	0.304	-0.062-0.670	0.012
		Neg	1	42	43			
	III	Pos	5	9	14	0.209	-0.083—-0.59	0.131
		Neg	6	31	37			
	IV	Pos	12	8	20	0.141	-0.123—-0.405	0.301
		Neg	14	17	31			
	V	Pos	2	7	9	0.030	-0.254—-0.314	0.828
		Neg	8	34	42			

CI=Confidence interval Pos= Positive, Neg= Negativexs.

In stage II, kappa (κ) of 0.304 represented a fair strength of agreement and a p value of 0.012(p<0.05) which was statistically significant. In stage III, kappa was 0.209 indicating none to slight agreement and a p value of 0.131 (p>0.05); stage IV kappa (κ) was 0.141(slight agreement) and p value of 0.301 (p>0.05). In stage V; kappa (κ) of 0 .030 represented poor agreement and a p value of 0.828(p>0.05).

Overall kappa analysis showed only poor to fair strength of agreement for different stages of lupus nephritis(0.030-0.304). Although only in stage II, p-value was <0.05 indicating statistically significant that there is a relation between clinical renal parameters with renal biopsy while in rest of other stages it showed no significant relation.

In addition, further analysis revealed that out of 8 patients considered to have stage II lupus nephritis on their laboratory parameters, when compared to their histopathology results, it was only matched with 2 patients (25%) while 3 patients (37.5%) were in stage V, 2 (25%)were in stage III and 1 patient (12.5%) was in stage IV on biopsy report.

Similarly, for stage III lupus nephritis, 05 (35.7%) out of 14 patients, laboratory and histopathology results were matched. One patient (7.14%) was found to have stage II, 7 patients (50%) were in stage IV and 1 patient (7.1) was in stage V lupus nephritis on histopathology results.

There were 20 patients labeled stage IV lupus nephritis on laboratory parameters. Among them 12 (60%) patients histopathology report found the same stage while 3 patients (15%) were in stage III, 4 patients (20%) were in stage V and 1 patient (5%) labeled stage VI on biopsy report.

Nine patients labeled stage V on laboratory parameters were compared with their histopathology findings, only 2 (22.2%)patients results were matched while 6(66.6%) were in stage IV and 1 (11.1%) was in stage III.

This indicates the fact that it is inadequate to rely on laboratory impression solely for starting treatment and renal biopsy should be performed in all cases of SLE having any urinary abnormality.

## DISCUSSION

Lupus nephritis has varied clinical presentation. Urinary abnormalities especially proteinuria is the cardinal feature of the disease. 25% of patients may have abnormal urine sediment at the time of diagnosis and 60% will develop some abnormality during their illness.^12^ The decision of when to perform renal biopsy in lupus nephritis is variable. Some studies recommend biopsy when patients have > 500 mg protein/24hours^13^ in the absence of renal failure, while others recommend biopsy only when proteinuria is >1000 mg/24 hours and abnormal urine sediment.^14^

Several case series have demonstrated that significant renal damage may be present without clinical signs of renal involvement in advanced staging of lupus nephritis(stage III, IV).^2^,^3^,^15^ Since early diagnosis can change the renal outcome,^16^,^17^ it is imperative that correct diagnosis be made. Our study reinforces this concept as 17/51 (33.3%) of patients, thought to have stages that did not require aggressive immunosuppressant therapy actually had advanced stages proven on renal biopsy that warranted institution of early immunosuppressant therapy. Our results are similar to results reported by Lisa Christopher et al. in which 55% of patients with low grade proteinuria had advanced Stage 3 or 4.^18^

Our study is an eye opener, as patients with SLE are usually treated by Rheumatologists and they follow clinical renal parameters to diagnose staging of lupus nephritis. In a survey done at JHUSOM, involving rheumatologists 37% would not refer the patient to a nephrologist with proteinuria <1000mg/24 hours and 17% would not refer even in the presence of hematuria.^19^

### Limitations of the study

We did not considered C3 levels and anti-ds DNA titers as they may influence the clinical staging, since studies have suggested that these parameters are indicative of proliferative stages. In addition, it is limited by a small sample size, but still the results are significant and need further studies as it may have a very important role in preventing long term renal outcome.

## CONCLUSION

Staging of lupus nephritis done on basis of laboratory findings did not correlate well with the underlying histopathological staging, and therefore, renal biopsy is an essential tool to approach lupus nephritis in order to provide timely and appropriate treatment to patients. We advocate early renal biopsy in all patients with LN, regardless of degree of proteinuria or renal function.
